# The Crustal Dynamics and Its Geological Explanation of the Three-Dimensional Co-Seismic Deformation Field for the 2021 Maduo *M*_S_7.4 Earthquake Based on GNSS and InSAR

**DOI:** 10.3390/s23083793

**Published:** 2023-04-07

**Authors:** Xiaobo Li, Yanling Chen, Xiaoya Wang, Renwei Xiong

**Affiliations:** 1 Shanghai Astronomical Observatory, Chinese Academy of Sciences, Shanghai 200030, China; 2 School of Astronomy and Space Science, University of Chinese Academy of Sciences, Beijing 100049, China; 3 Institute of Earthquake Forecasting, China Earthquake Administration, Beijing 100036, China; 4 Shanghai Key Laboratory of Space Navigation and Positioning Techniques, Shanghai Astronomical Observatory, Chinese Academy of Sciences, Shanghai 200030, China

**Keywords:** integration of GNSS and InSAR, three-dimensional co-seismic deformation, pre-existing fault, adaptive method, Maduo *M*_S_7.4 earthquake

## Abstract

Three-dimensional deformation is an important input to explore seismic mechanisms and geodynamics. The GNSS and InSAR technologies are commonly used to obtain the co-seismic three-dimensional deformation field. This paper focused on the effect of calculation accuracy caused by the deformation correlation between the reference point and the points involved in the solution, to build a high-accuracy three-dimensional deformation field for a detailed geological explanation. Based on the variance component estimation (VCE) method, the InSAR LOS, azimuthal deformation, and the GNSS horizontal and vertical deformation were integrated to solve the three-dimensional displacement of the study area in combination with the elasticity theory. The accuracy of the three-dimensional co-seismic deformation field of the 2021 Maduo *M*_S_7.4 earthquake obtained by the method proposed in this paper, was compared with that obtained from the only InSAR measurements obtained using a multi-satellite and multi-technology approach. The results showed the difference in root-mean-square errors (RMSE) of the integration and GNSS displacement was 0.98 cm, 5.64 cm, and 1.37 cm in the east–west, north–south and vertical direction respectively, which was better than the RMSE of the method using only InSAR and GNSS displacement, which was 5.2 cm and 12.2 cm in the east–west, north–south, and no vertical direction. With the geological field survey and aftershocks relocation, the results showed good agreement with the strike and the position of the surface rupture. The maximum slip displacement was about 4 m, which was consistent with the result of the empirical statistical formula. It was firstly found that the pre-existing fault controlled the vertical deformation on the south side of the west end of the main surface rupture caused by the Maduo *M*_S_7.4 earthquake, which provided the direct evidence for the theoretical hypothesis that large earthquakes could not only produce surface rupture on seismogenic faults, but also trigger pre-existing faults or new faults to produce surface rupture or weak deformation in areas far from seismogenic faults. An adaptive method was proposed in GNSS and InSAR integration, which could take into account the correlation distance and the efficiency of homogeneous point selection. Meanwhile, deformation information of the decoherent region could be recovered without interpolation of the GNSS displacement. This series of findings formed an essential supplement to the field surface rupture survey and provided a novel idea for the combination of the various spatial measurement technologies to improve the seismic deformation monitoring.

## 1. Introduction

The three-dimensional deformation field caused by any geological hazard is an important parameter in the study of crustal movement characteristics, which implies certain geodynamics. Accurately acquiring the complete regional three-dimensional crustal deformation field is an important research direction for the dynamics of regional crustal deformation and understanding its geohazard process. The rapid development of geodetic measurement technologies, such as InSAR (Interferometric Synthetic Aperture Radar) and GNSS (Global Navigation Satellite System), provides an opportunity to acquire an accurate regional three-dimensional crustal deformation field. The InSAR has the advantages of all-weather, all-day, high-spatial resolution, while it only provides one-dimensional deformation information, so that the monitoring of true crustal deformation dynamics is restricted. The GNSS can provide high-precision three-dimensional deformation information [1], but its spatial resolution is influenced by the density of GNSS stations so that the obtained deformations are discrete, which also limits the study of regional crustal dynamics.

Integrating the advantages of these two observations to obtain high-accuracy and high-spatial-resolution three-dimensional deformation, is a subject that has been explored. Since Guemundsson [2] proposed the concept of combining GPS and INSAR to study crustal deformation, the studies [3,4,5,6,7,8,9,10,11,12,13] have focused on optimizing the Gibbs energy equation for acquiring three-dimensional deformation. They required interpolating GNSS data to the pixel positions of InSAR images [14] and establishing nonlinear equations without considering the correlation between each adjacent point. However, the interpolation itself had errors [15], which would become greater, especially when the abrupt deformation happened, such as earthquakes, volcanoes, and so on. It is not suitable for high accuracy deformation monitoring.

Guglielmino [16] proposed a non-interpolation method for reconstructing three-dimensional deformation fields by integrating GNSS and InSAR, namely, the SISTEM (simultaneous and integrated strain tensor estimation from geodetic and satellite deformation measurements), which took into account the spatial correlation between adjacent points, and the linear relationship between strain and displacement based on the elasticity theory. Guglielmino [16,17,18] solved the area of interest three-dimensional displacement field by the weighted least square (WLS) adjustment by using the GNSS discrete three-dimensional displacements around a reference point and the DInSAR line of sight (LOS) direction displacement at the reference point, which did not consider the correlation between the adjacent points and the reference point. This method could not integrate InSAR and GNSS data in the decoherence regions due to the absence of InSAR data. Luo et al. [19,20] increased the InSAR LOS direction displacement around the reference point and developed the ESISTEM (extended simultaneous and integrated strain tensor estimation from geodetic and satellite deformation measurements) method, which used all GNSS horizontal displacement. The above-mentioned methods did not consider the deformation correlation between the GNSS stations and the reference point. Furthermore, the vertical deformation accuracy of GNSS measurement reached the millimeter level, and InSAR azimuthal direction displacement was sensitive to the north–south deformation, which could contribute to a constraint on the accuracy of the three-dimensional deformation.

The high construction cost and strict setting requirements determine the uneven distribution density of GNSS stations. In many cases, only InSAR was used to obtain three-dimensional deformation [21,22,23,24,25,26,27,28,29,30] by integrating displacements obtained from different orbits (ascending and descending) and different InSAR techniques, such as the Differential Interferometric Synthetic Aperture Radar (DInSAR) [31], the multiple aperture interferometry (MAI) [32], the pixel offset-tracking (POT) [33], the burst overlap interferometry (BOI) [34]. However, the InSAR measurements were of relative displacement, which should be constrained by GNSS absolute displacement to obtain the absolute three-dimensional deformation in higher accuracy.

Taking the 2021 Maduo *M*_S_7.4 earthquake in Qinghai, China, which occurred in the Bayan Har block as an example, we collected more than 100 GNSS stations in the surrounding area of interest, and the InSAR co-seismic one-dimensional deformation. Based on the elasticity theory [35], we established the linear equation between the strain and displacement pixel by pixel, in which the GNSS stations within the correlation distance were automatically searched, and the homogeneous InSAR pixels involved in the solution were adaptively adjusted. We obtained an accurate co-seismic three-dimensional deformation field of the area of interest, including the deformation of decoherence region, by the variance component estimation (VCE) weighting [36,37,38,39,40,41,42]. Furthermore, we evaluated the accuracy of the calculated three-dimensional displacement field and analyzed the deformation characteristics in the east–west, north–south, and vertical directions, respectively. The crustal dynamics and its geological explanation were analyzed by combining the solution with the field geological survey and relocation. 

## 2. Study Area and Data Processing

### 2.1. Study Area

**Figure 1:**
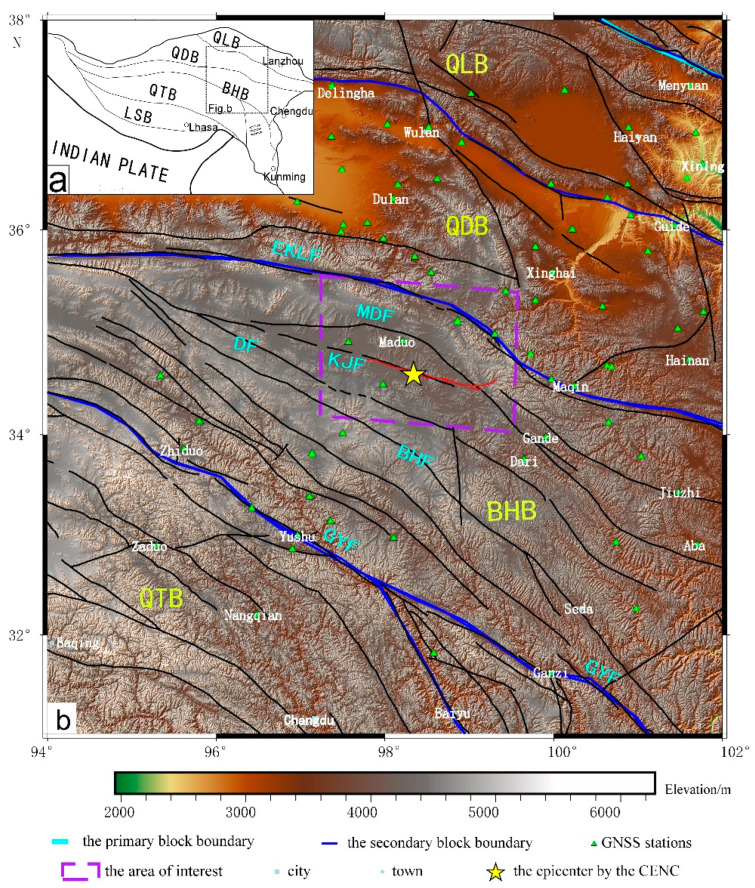
The tectonic location of the 2021 Maduo *M*_S_7.4 earthquake and distribution of the used GNSS station in the study area. (**a**) is the tectonic unit of the Qinghai-Tibet Plateau and (**b**) is the specific tectonic location and active fault of the black box in (**a**): the green-yellow font represents the abbreviated name of the secondary tectonic block, the light blue font represents the abbreviated name of the main fault, the cyan line represents the primary block boundary, the blue line represents the secondary tectonic block boundary, and the black line is the active fault divided by Deng et al. [44]. The yellow pentagram represents the epicenter location of the 2021 Maduo MS7.4 earthquake according to the China earthquake networks center (CENC), the green triangle represents the GNSS stations near the area of interest, which shows some stations overlap because of close ranges, the purple dotted frame is the area of interest, and the red line is the surface rupture caused by the earthquake, the light blue square and dot are city and town, respectively. BHB—Bayan Har Block, QLB—Qilian Block, QDB—Qiadam Block, GTB—Qiangtang Block, LSB—Lhasa Block, EKLF—East Konglun Fault, MDF—Maduo Fault, KJF—Kunlongshankou–Jiangcuo Fault, DF—Dari Fault, BHF—Bayan Har Mountain piedmont Fault, GYF—Ganzi-Yushu–Xianshuihe Fault.

An *M*_S_7.4 earthquake occurred in Maduo County, Luozhou, Qinghai, China on 22 May 2021, whose epicenter located at 34.59° N, 98.34° E, and the source depth was 17 km (http://www.ceic.ac.cn/history, accessed on 12 July 2021), which was the largest earthquake after the 2008 Wenchuan *M*_W_ 7.9 earthquake in China. The Maduo *M*_S_7.4 earthquake occurred on the Bayan Har block of the Qinghai–Tibet Plateau secondary tectonic block, 70 km south of the Toso Lake section of the East Kunlun Fault. The Bayan Har block had a very complex tectonic background, and was surrounded by the Ganzi–Yushu–Xianshuihe Fault to the south, the Longmenshan Fault to the east, the East Kunlun Fault to the north, the Maergaichaka Fault and the Altyn Fault to the west. The southern side of the Bayan Har block was pushed by the Indian plate to the northeast and was blocked by the Tarim Basin, which caused material flow and formed the uplift deformation on the Qinghai–Tibet Plateau. Due to the blockage of the eastern Erdos block and the Sichuan Basin, the Qinghai–Tibet Plateau as a whole continued escaping to the southeast, and the geotectonic movements were active and easily induced large earthquakes in the block. In recent years, many strong earthquakes have occurred on the Bayan Har block, such as the 2001 Kunlun Mountain Pass West *M*8.1 earthquake, the 2008 Wenchuan *M*8.0 earthquake, the 2010 Yushu *M*7.1 earthquake, the 2013 Lushan *M*7.0 earthquake, and the 2017 Jiuzhaigou *M* 7.0 earthquake, which indicated that the block was in a strong active state. It was necessary to pay more attention to geological hazards in this area. The 2021 Maduo *M*_S_7.4 earthquake occurred on a secondary fault that was approximately parallel to the East Kunlun Fault [43]. However, the seismic hazard of this secondary fault had been lacking special attention. Figure 1 shows the tectonic unit and the location of the 2021 Maduo *M*_S_7.4 earthquake and the used data in the area of interest.

So far, the seismogenic structure and deformation characteristics of the Maduo *M*_S_7.4 earthquake had been researched by various means [45,46,47,48,49,50,51,52,53], which showed that this earthquake was a special and complex left-lateral slip earthquake. Unlike the previous recognition that the main surface rupture was located at the epicenter, no obvious surface rupture had been found near the epicenter according to the field investigation; the obvious surface rupture was far away from the epicenter, and many fissures and extrusion bulges in very different directions were near the surface rupture [45,54], which showed the earthquake was a complex event. As the most intuitive manifestation of an earthquake, the accurate measurement of the three-dimensional deformation contributes to a better understanding of crustal dynamics and seismic triggering mechanisms. 

The GNSS stations from different organizations provided the research conditions for the discrete three-dimensional deformation in the Qinghai–Tibet Plateau. However, the regional co-seismic three-dimensional deformation could not be solved simply by interpolating GNSS data. As a complement for the low spatial resolution of GNSS stations, InSAR measurement could be used to obtain high spatial resolution one-dimensional deformation of the study area. 

### 2.2. GNSS Data Estimated the Three-Dimensional Deformation

Using the GAMIT/GLOBK software, the time series of high accuracy GNSS three-dimensional deformation could be obtained before and after the earthquake. This paper applied the continuous GNSS observation stations from different sources, including the Crustal Movement Observation Network of China, the provincial earthquake agency, enterprises, and more than 200 IGS stations uniformly distributed worldwide [55]. 

Firstly, all the stations used were loosely constrained, and their daily solutions by the GAMIT software were obtained, where the baseline solution used the RELAX mode, the satellite elevation cutoff angle was 10°, the atmospheric zenith wet delay was solved every 2 h, and mapped to the satellite by the GMF mapping function. All files, including the receiver antenna file, the satellite antenna file, the receiver and satellite antenna phase center models, the solid tide model, the solar ephemeris table, the lunar ephemeris table, and the ocean tide model, were updated. The daily solutions were combined with the calculated global distribution of 200 IGS stations. then, we chose some global, even, stable, and self-consistent IGS stations as constraints, and performed a seven-parameter transformation to tie the above solution to the International Terrestrial Reference Frame 2014 (ITRF14) by the GLOBK software. We could obtain the accurate three-dimensional coordinates time series under the ITRF 2014.

To avoid the after-slip effect and increase the GNSS measurement reliability, the co-seismic displacement of GNSS stations before and after the earthquake was chosen based on the principle that the ratio of displacement to error was greater than 1. Some data after the earthquake was removed, so we used selected data, including 3 days before the earthquake and 1~3 days after the earthquake, to calculate the co-seismic deformation. The station time series were averaged, respectively, and the differential before and after the earthquake, the errors in each direction could be obtained by the error propagation law. 

**Figure 2:**
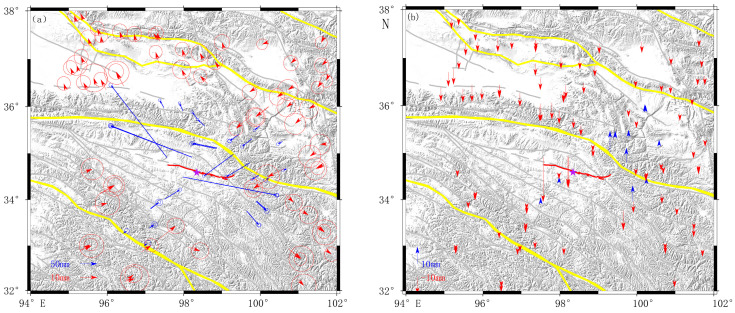
The co-seismic displacement obtained by GNSS data. The yellow lines are the secondary tectonic block boundary, (**a**) represents the co-seismic displacement in horizontal direction, the blue and the red arrows represent the different displacement ratio, and (**b**) represents the co-seismic displacement in the vertical direction. The blue represents the uplift displacement, and the red represents the subsidence displacement: the red circle represents the error ellipse, the purple star represents the epicenter of the Maduo *M*_S_7.4 earthquake, the red curve near the epicenter represents.

In Figure 2, we can see that more displacements were near the epicenter. In Figure 2a, the horizontal co-seismic displacement was symmetrically distributed in four quadrants. Moreover, some stations’ horizontal co-seismic displacement directions were parallel to the seismogenic fault, which indicated it was a left-lateral strike-slip earthquake. In Figure 2b, we could see an uplift to the west and subsidence to the east on the northern side of the seismogenic fault, and subsidence and small displacement could be found on the southern side of the fault, which indicated it was uneven deformation in the vertical direction.

### 2.3. InSAR Data Estimated the One-Dimensional Deformation

InSAR was the space-to-earth observation combining synthetic aperture radar (SAR) technology with interferometric technology. The SAR satellites are in an almost polar orbit around the north and south poles of the Earth, thus it was very insensitive to the north–south (NS) deformation of InSAR LOS direction, while the azimuthal direction could make up for the shortcoming of the InSAR LOS direction. The DInSAR technique could acquire the LOS deformation, and the MAI and POT techniques could acquire the LOS and azimuthal deformation, in which the azimuthal accuracy of the MAI was significantly better than that of the POT in areas with higher coherence, while the POT could resist decoherence and obtain the fault trace, which all belong to the InSAR measurement.

The SAR satellite provided two different geometry SAR images by ascending and descending orbits, respectively. The research shows [56,57] that it was of benefit to improving the accuracy of the three-dimensional deformation field by both ascending and descending at the same time. That was, the measured deformation should include ascending LOS, descending LOS, ascending azimuth, and descending azimuth. 

The European Space Agency (ESA) launched two C-band SAR satellites, the Sentinel-1A satellite, and Sentinel-1B satellite, in April 2014 and April 2016, respectively. The revisit period was 12 days for a single satellite, while the revisit period could be shortened to 6 days for two satellites. The Sentinel-1 observation data could be downloaded freely from ESA official website (https://scihub.copernicus.eu/dhus/#/home, accessed on 12 July 2021) by registered users, and the corresponding precise orbit file can be downloaded freely about 21 days after measurement on the other website (https://scihub.copernicus.eu/gnss/#/home, accessed on 12 July 2021). We downloaded the Sentinel-1A/B interferometric wide (IW) swath mode single look complex (SLC) data before and after the 2021 Maduo *M*_S_7.4 earthquake, as well as the precise orbit files at the corresponding time. The specific information is shown in Table 1.

**Figure 3:**
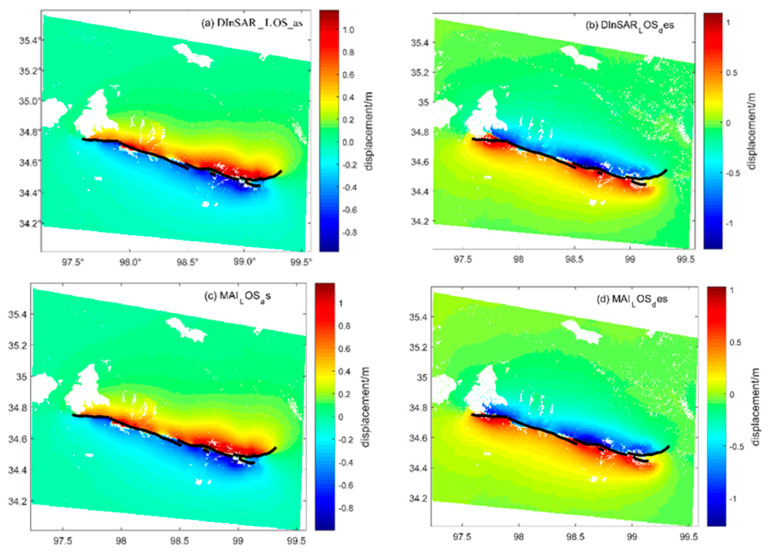

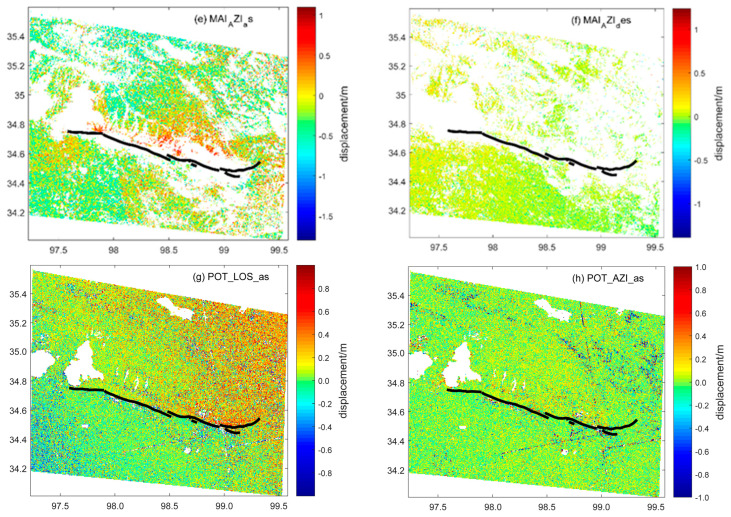
The LOS and azimuthal deformations obtained by the DInSAR, MAI and POT, the *x*-axis represents the east longitude (E), unit is the degree; the *y*-axis represents the north latitude (N), unit is the degree; (**a**) DInSAR_LOS_as; (**b**) DInSAR_LOS_des; (**c**) MAI_LOS_as; (**d**) MAI_LOS_des; (**e**) MAI_AZI_as; (**f**) MAI_AZI_des; (**g**) POT_LOS_as; (**h**) POT_AZI_as. For example, DInSAR_LOS_des indicated the descending LOS deformation obtained by DInSAR technique; the black line represents the surface rupture in field, which is agree with the InSAR measurement results.

In this paper, the InSAR measurements, including the DInSAR, MAI and POT techniques, were used to obtain interferograms for the ascending and descending Sentinel-1A/B satellites data, respectively. In the processing, the STRM DEM [58] with 30 m resolution was used to eliminate terrain error effect, the multi-looking was carried out 2:10 by azimuth resolution and range resolution to reduce the speckle noise of the SAR images and improve signal-to-noise ratio, the phase unwrapping was carried out by Goldstein filtering and the minimum cost flow solved the problem of the phase ambiguity, the precision orbit files could be used to re-flatten and eliminate orbital errors by selecting the ground control points and the polynomial refinement method, then the geocoding was carried out to obtain the LOS and azimuthal co-seismic deformation in the WGS84 coordinate system of the 2021 Maduo *M*_S_7.4 earthquake. The results were expressed as DInSAR_LOS_des, DInSAR_LOS_as, MAI_LOS_des, MAI_LOS_as, MAI_AZI_des, MAI_AZI_as, POT_LOS_des, POT_ LOS_as, POT_AZI_des, and POT_AZI_as. For example, DInSAR_LOS_des represented the descending LOS deformation obtained by the DInSAR technique, and MAI_AZI_as represented the ascending azimuthal deformation obtained by the MAI technique. The specific co-seismic deformation results from different techniques are shown in Figure 3.

The azimuthal accuracy of the POT technique was 1/30~1/10 of the pixel azimuthal resolution used. However, the azimuthal resolution was slightly low in the Sentinel-1 satellite, so that a lot of noise occurred in the deformation result from the POT. In this paper, the POT only was used to delineate the fault traces and eliminated non-homogeneous points by its resisting decoherence advantage. For the InSAR azimuthal deformation, we only used the MAI azimuthal direction deformation, which was more accurate than the POT. 

## 3. Methodology

### 3.1. Multi-Source Integrating the Three-Dimensional Deformation

According to the respective advantages of the GNSS and InSAR, we built the high-accuracy and high-spatial-resolution regional three-dimensional deformation fields, and provided the foundation for detailed analysis of the dynamic process in the area.

Quantitative description in seismology is based on solid continuum mechanics and the infinitesimal and homogeneous unit, often known as elasticity mechanics or elasticity theory. The displacement between a point r and the reference point $r_{0}$ was $\Delta d=r-r_{0}$. Based on the elasticity theory, strain was the spatial gradient of displacement, and was a relative value. The relation between the displacement and the strain could be expressed as $\varepsilon =\frac{r-r_{0}}{r}$.

When a geodynamic process occurs, such as earthquakes or volcanoes, and deforms a portion of the earth’s surface, the surface is considered to be a uniform strain [16]. An arbitrary point P locates the position $\left(x_{e}^{0},x_{n}^{0},x_{u}^{0}\right)$, and its three-dimensional displacement $\left(d_{e}^{0},d_{n}^{0},d_{u}^{0}\right)$ comprises unknown components, and the surrounding N points’ position $\left(x_{e}^{i},x_{n}^{i},x_{u}^{i}\right)$ and the three-dimensional displacement $\left(d_{e}^{i},d_{n}^{i},d_{u}^{i}\right)$ are known (i=1,2,…,N). For the infinitesimal deformation, we could perform the Taylor expansion for each of the three components without taking into account the second orders and the above terms. Thus, the equation for the linear relationship between strain and displacement is as follows.

(1) $$d^{i}=H\cdot \Delta x^{i}+d^{0}$$

In which the $\Delta x_{}^{i}=x_{}^{i}-x_{}^{0}$ represents the vector distance between the point i and point P in the corresponding east (E), north (N), and vertical (U) directions, which are needed to convert the coordinates of all points to the same geodetic coordinate system. The H represented the displacement gradient tensor, which could be expressed by the symmetric strain tensor ε and anti-symmetric rigid body rotation tensor ω [16].

(2) $$H=\varepsilon +\omega =\left[\begin{array}{ccc} \varepsilon_{11} & \varepsilon_{12} & \varepsilon_{13}\\ \varepsilon_{21} & \varepsilon_{22} & \varepsilon_{23}\\ \varepsilon_{31} & \varepsilon_{32} & \varepsilon_{33} \end{array}\right]+\left[\begin{array}{ccc} 0 & -\omega_{3} & \omega_{2}\\ \omega_{3} & 0 & -\omega_{1}\\ -\omega_{2} & \omega_{1} & 0 \end{array}\right]$$

By bringing the Equation (2) into Equation (1), it could be expressed as

(3) $$\left[\begin{array}{c} d_{e}^{i}\\ d_{n}^{i}\\ d_{u}^{i} \end{array}\right]=\left[\begin{array}{ccc} \varepsilon_{11} & \varepsilon_{12}-\omega_{3} & \varepsilon_{13}+\omega_{2}\\ \varepsilon_{21}+\omega_{3} & \varepsilon_{22} & \varepsilon_{23}-\omega_{1}\\ \varepsilon_{31}-\omega_{2} & \varepsilon_{32}+\omega_{1} & \varepsilon_{33} \end{array}\right]\cdot \left[\begin{array}{c} \Delta x_{e}^{i}\\ \Delta x_{n}^{i}\\ \Delta x_{u}^{i} \end{array}\right]+\left[\begin{array}{c} d_{e}^{0}\\ d_{n}^{0}\\ d_{u}^{0} \end{array}\right]$$

In which, the unknown matrix was expressed as follows.

(4) $$X=\left[\begin{array}{cccccccccccc} d_{e}^{0} & d_{n}^{0} & d_{u}^{0} & \varepsilon_{11} & \varepsilon_{12} & \varepsilon_{13} & \varepsilon_{22} & \varepsilon_{23} & \varepsilon_{33} & \omega_{1} & \omega_{2} & \omega_{3} \end{array}\right]$$

The coefficient matrix was as follows.

(5) $$A_{SM}=\left[\begin{array}{cccccccccccc} 1 & 0 & 0 & \Delta x_{e}^{i} & \Delta x_{n}^{i} & \Delta x_{u}^{i} & 0 & 0 & 0 & 0 & \Delta x_{u}^{i} & -\Delta x_{n}^{i}\\ 0 & 1 & 0 & 0 & \Delta x_{e}^{i} & 0 & \Delta x_{n}^{i} & \Delta x_{u}^{i} & 0 & -\Delta x_{u}^{i} & 0 & \Delta x_{e}^{i}\\ 0 & 0 & 1 & 0 & 0 & \Delta x_{e}^{i} & 0 & \Delta x_{n}^{i} & \Delta x_{u}^{i} & \Delta x_{n}^{i} & -\Delta x_{e}^{i} & 0 \end{array}\right]$$

Then,

(6) $$\left[\begin{array}{c} d_{e}^{i}\\ d_{n}^{i}\\ d_{u}^{i} \end{array}\right]=A_{SM}^{i}\cdot X$$

For the GNSS measurement, the GNSS stations discrete co-seismic three-dimensional deformation can be directly obtained by displacement difference before and after an earthquake. It can be expressed as follows.

(7) $$\left[\begin{array}{c} L_{e}^{i}\\ L_{n}^{i}\\ L_{u}^{i} \end{array}\right]=A_{geo}^{i}\cdot d^{i}=\left[\begin{array}{ccc} 1 & 0 & 0\\ 0 & 1 & 0\\ 0 & 0 & 1 \end{array}\right]\cdot \left[\begin{array}{c} d_{e}^{i}\\ d_{n}^{i}\\ d_{u}^{i} \end{array}\right]$$

$L_{e}^{i}$, $L_{n}^{i}$, $L_{u}^{i}$ were the GNSS stations deformations in east–west, north–south and vertical directions, respectively.

For the InSAR measurement, only one-dimensional deformation could be obtained in the azimuthal or LOS direction, and needed to be decomposed into three-dimensional direction by a certain formula. The relationship between three-dimensional and one-dimensional displacement of the same point was as follows.

(8) $$\left[\begin{array}{c} L_{losdes}^{i}\\ L_{losas}^{i}\\ L_{azides}^{i}\\ L_{azias}^{i} \end{array}\right]=A_{geo}^{i}\cdot d^{i}=\left[\begin{array}{ccc} a_{losdes}^{i} & b_{losdes}^{i} & c_{losdes}^{i}\\ a_{losas}^{i} & b_{losas}^{i} & c_{losas}^{i}\\ a_{azides}^{i} & b_{azides}^{i} & c_{azides}^{i}\\ a_{azias}^{i} & b_{azias}^{i} & c_{azias}^{i} \end{array}\right]\cdot \left[\begin{array}{c} d_{e}^{i}\\ d_{n}^{i}\\ d_{u}^{i} \end{array}\right]$$

In which

$$\begin{array}{ll} a_{losdes}^{i}=-\sin\left(\theta_{des}^{i}\right)\cdot \sin\left(\alpha_{des}^{i}-3\pi /2\right)\\ b_{losdes}^{i}=-\sin\left(\theta_{des}^{i}\right)\cdot \cos\left(\alpha_{des}^{i}-3\pi /2\right), & c_{losdes}^{i}=\cos\left(\theta_{des}^{i}\right)\\ a_{losas}^{i}=-\sin\left(\theta_{as}^{i}\right)\cdot \sin\left(\alpha_{as}^{i}-3\pi /2\right)\\ b_{losas}^{i}=-\sin\left(\theta_{as}^{i}\right)\cdot \cos\left(\alpha_{as}^{i}-3\pi /2\right), & c_{losas}^{i}=\cos\left(\theta_{as}^{i}\right)\\ a_{azides}^{i}=-\cos\left(\alpha_{des}^{i}-3\pi /2\right)\\ b_{azides}^{i}=\sin\left(\alpha_{des}^{i}-3\pi /2\right), & c_{azides}^{i}=0.0\\ a_{azias}^{i}=-\cos\left(\alpha_{as}^{i}-3\pi /2\right)\\ b_{azias}^{i}=\sin\left(\alpha_{as}^{i}-3\pi /2\right), & c_{azias}^{i}=0.0 \end{array}$$

$L_{losas}^{i}$, $L_{losdes}^{i}$, $L_{azias}^{i}$, $L_{azides}^{i}$ represent the InSAR LOS and azimuthal displacement of the ascending and descending by the different measurement techniques, respectively. $\alpha_{as}^{i}$, $\alpha_{des}^{i}$, $\theta_{as}^{i}$, $\theta_{des}^{i}$ represented the pixel i heading angles and elevation angles of the ascending and descending orbits, respectively. Equation (8) was a unified expression of the orbit and direction, which could be obtained by different techniques, for example, the $L_{losas}^{i}$ was the ascending LOS displacement which may be obtained by the DInSAR or the MAI. Then, the above corresponding relationship of the five deformation types (DInSAR_LOS_des, DInSAR_LOS_as, MAI_LOS_des, MAI_LOS_as, and MAI_AZI_as) involved in the solution can be brought in Equation (8).

According to Equations (6)–(8), the relationship between the observations and the calculated values was as follows.

(9) $$L=A_{geo}^{i}\cdot d^{i}+\varepsilon =A_{geo}^{i}\cdot A_{SM}^{i}\cdot X+\varepsilon =A\cdot X+\varepsilon$$

The error equations could be listed as follows.

(10) v=A⋅X−L

Establishing the observation equation involved two critical steps: the selection of the surrounding observations that were related to the reference point, and the integration of the data by an appropriate weighting allocation. We needed to determine the deformation correlation distance of each reference point and adjust the relevant window size around the reference point, in order to establish the observation equations with the correlation conditions. On this basis, determining the reasonable weights for various observations was essential to obtaining high accuracy deformation results in the process of integrating different measurement technologies. 

### 3.2. Adaptive Selection of the Relevant Points 

The research showed that [25] the number of observations involved in the solution had a great influence on the calculation accuracy and efficiency. The larger the window size, the more pixels involved in the solution, the less the difference the root-mean-square error (RMSE) between the calculated result and GNSS displacement in the three components, and the three-dimensional deformation was more accurate. However, the deformation correlation between the observation and the reference point became weakened or even irrelevant with the increase in the window size, which would not satisfy the requirement of the same deformation model. Since it had an impact on the deformation correlation and the computational efficiency, the window size should have a certain constraint threshold. To this end, we established a variogram [59,60] by the InSAR pixels to determine the correlation distance in the area of interest. The geostatistical approach used in references [59,60] demonstrated that it was the exponential relationship between the covariance and distance. The equation was as follows.

(11) $$C\left(h\right)=C_{0}\cdot e^{-\frac{h}{s}}$$

where C(h) was the covariance function [56] between pixels with a distance h in the study area, $C_{0}$ was the variance of the study area, and s was the unknown correlation distance and proportional to h. According to the relationship between the pixel spatial resolution and the distance, the h and s could be directly expressed as the window size at the same time.

When more than 200 pixels were involved in the solution, the RMSE tended to be steady [25]. We linearized formula (11) and established the observation equations by the 15 × 15 pixels as the initial window size. The correlation distance threshold could be determined by the least squares fitting with the window sizes being gradually increased, for each technological observation. To guarantee that each technological observations could fulfill the correlation distance, the final window size threshold was the minimum of the correlation distance thresholds of the various measurement technologies. The window size was automatically expanded until that more than 200 pixels were involved in the solution. 

The heterogeneous points near the fault must be considered in the process of adaptively adjusting the window sizes, due to the deformation characteristics being different on both sides of the earthquake fault. To search heterogeneous points around the reference point and remove them, we obtained the specific earthquake fault trace and the boundary of the heterogeneous points, due to the POT being insensitive to decoherence. It was different from the empirical approach that all GNSS stations were involved in the solution; we chose the GNSS stations within the correlation distance in order to ensure the deformation correlation between the used observations and the reference point. It meant that the number of the GNSS stations and the InSAR pixels involved in the solution were variable pixel by pixel.

After determining the observations involved in the solution by adaptively adjusting the window size, we could establish the error equation. We considered weighting reasonably for the different observations.

### 3.3. Integrating Data Based on the VCE Weighting

The observations were classified according to their measurement accuracy. The GNSS observations were weighted as two types of observations, due to the different measurement accuracy in the horizontal and vertical directions. InSAR observations were weighted according to the directions and the orbit from different measurement technologies. Each type of observations was considered to be independent. 

Firstly, the prior variances reciprocal was as the initial weights. For the GNSS measurement, the prior variances were the error square in the corresponding directions. For InSAR measurement, the prior variances were calculated by using the pixels within the adaptive window size base on the ergodicity assumption; the formula is as follows.

(12) $$\sigma_{0j}^{2}=\frac{\sum_{i=1}^{i=k_{j}}{\left(d_{j}^{i}-{\bar{d}}_{j}\right)}^{2}}{k_{j}-1}$$

where $\sigma_{0j}^{2}$ represented the prior variance of the type j observations, j represented the type j observations, $d_{j}^{i}$ represented the pixel i deformation in the type j observations, $k_{j}$ represented the total number of the type j observations within the window size, ${\bar{d}}_{j}$ represented the average of the $k_{j}$ pixels.

After estimating the unit weight variance of each type of observation by the VCE method, the corresponding new weight was given by iterating the initial weight, that was as follows.

$$P_{1}{}^{\prime }=\frac{c}{\sigma_{1}{}^{2}\cdot P_{1}^{-1}},P_{2}{}^{\prime }=\frac{c}{\sigma_{2}{}^{2}\cdot P_{2}^{-1}},P_{3}{}^{\prime }=\frac{c}{\sigma_{3}{}^{2}\cdot P_{3}^{-1}},P_{4}{}^{\prime }=\frac{c}{\sigma_{4}{}^{2}\cdot P_{4}^{-1}},\;P_{5}{}^{\prime }=\frac{c}{\sigma_{5}{}^{2}\cdot P_{5}^{-1}},P_{6}{}^{\prime }=\frac{c}{\sigma_{6}{}^{2}\cdot P_{6}^{-1}},P_{7}{}^{\prime }=\frac{c}{\sigma_{7}{}^{2}\cdot P_{7}^{-1}}$$

where $P_{j}$ was the initial weight value of type j of observation, $P_{j}{}^{\prime }$ was the corresponding new weight that would participate in the new adjustment calculation, c represented a certain value generally, such as $\sigma_{j}^{2}$. The new unit weight variance could be obtained by the VCE method, iterating until the unit weight variances of all types of observations were approximately equal as follows.

$$\sigma_{1}^{2}\approx \sigma_{2}^{2}\approx \sigma_{3}^{2}\approx \sigma_{4}^{2}\approx \sigma_{5}^{2}\approx \sigma_{6}^{2}$$

In the weighting iteration process, the difference between the different types of unit weight variance should be less than a convergence threshold $\varepsilon^{2}$, which depended on the observation accuracy. Comprehensively considering the observation accuracy of the GNSS and InSAR measurement, for example, the GNSS measurement accuracy was millimeter or submillimeter, the DInSAR measurement accuracy was centimeter or millimeter, and the MAI measurement accuracy was decimeter or centimeter. We chose the unit weight variance convergence threshold of 0.0001 m^2^. That is, the end-of-iteration condition was satisfied $\sigma_{\max}^{2}-\sigma_{\min}^{2}<0.0001$. $\sigma_{\max}^{2}$ denoted the maximum value of the unit weight variances for the various types of observations, and $\sigma_{\min}^{2}$ denoted the minimum value.

## 4. Results and Analysis

**Figure 4:**
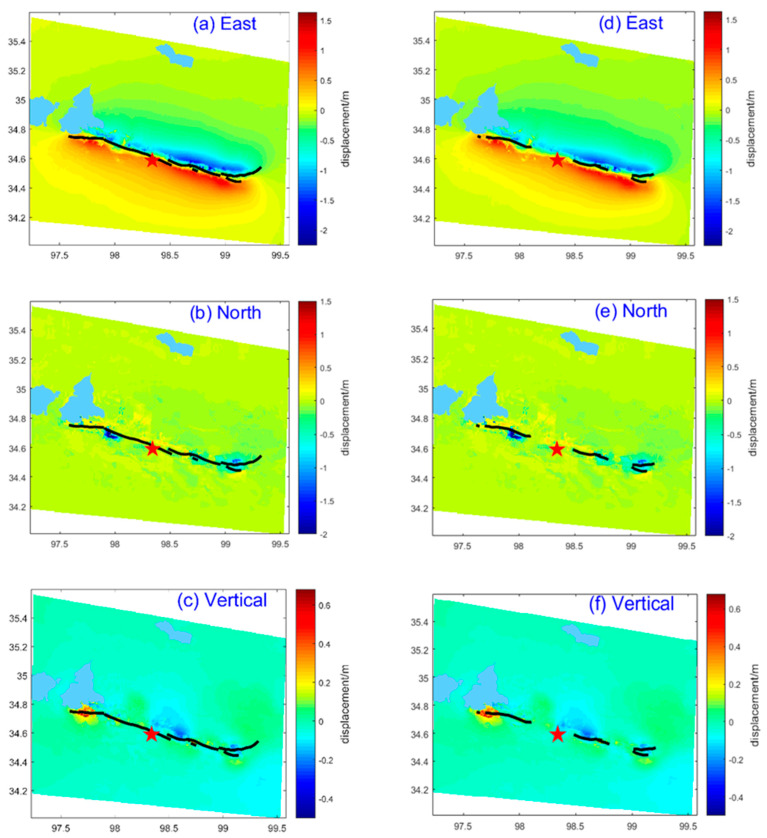
The co-seismic three-dimensional deformation field of the 2021 Maduo *M*_S_7.4 earthquake: (**a**,**d**) the displacement of the east–west direction; (**b**,**e**) the displacement of the north–south direction; (**c**,**f**) the displacement of the vertical direction. In Figure 4a–c, the black line is the fitted rupture from the filed investigation [44] and the InSAR deformation field in this study. In Figure 4d–f, the black line is the surface rupture from the fine interpretation from the unmanned aerial vehicle (UAV), the red star represents the epicenter of the Maduo *M*_S_7.4 earthquake.

In this paper, the three-dimensional surface deformation field in the hundreds of kilometers along the seismic fault near the epicenter was obtained by effectively integrating the GNSS and InSAR measurements. Figure 4 showed the regional co-seismic three-dimensional deformation field.

The strike of the earthquake fault was NWW and locally near EW. Since the angle between the strike of the earthquake fault and the east–west direction was small, the east–west deformation was dominant in the earthquake fault deformation. We considered that the east–west component result could indicate the earthquake fault movement mode. As shown in Figure 4, when the displacement was decomposed into three components, the displacement was large in the east–west direction with a wide range (See Figure 4a,d), and obvious left-lateral shear stress happened on both sides of the fault (the displacement to the west in the north of the earthquake fault, the displacement to the east in the south of the earthquake fault), which indicated that it was a typical left-lateral strike-slip earthquake. The deformations at both ends of the main surface rupture far away from the epicenter were larger than that near the epicenter. In the vertical, the deformation on both sides of the rupture was smaller and anti-symmetrically alternating between subsidence and uplift. These deformation characteristics were consistent with the results of the field geological survey [44,45,53] and were in agreement with the focal mechanism solution [61].

The field scientific investigation of the 2021 Maduo *M*_S_7.4 earthquake indicated [44] that no obvious main surface rupture had occurred near the epicenter (shown in Figure 4d–f), while there was secondary crack development in a wide range (mainly seismic fractures). Away from the epicenter, obvious main surface ruptures had been found along the eastern and western sides of the earthquake fault. Continuing eastward along the fault, no main surface rupture was found but some small secondary cracks (mainly seismic fractures), until the main surface rupture appeared at the northeast and southwest bifurcation phenomenon. However, at the western end of the main surface rupture, the rupture strike also shifted suddenly to a southern deflection. 

We overlaid the main surface rupture of the geological mapping with the three-dimensional deformation field. In Figure 4a–c, the black line was the fitted rupture from the filed investigation [44] and the InSAR deformation field in this study. In Figure 4d–f, the black line is the surface rupture from the fine interpretation from the unmanned aerial vehicle (UAV). The east–west directional deformation in Figure 4a was in good agreement with the rupture strike. The apparent displacement to the west on the north of the earthquake fault and to the east on the south, also indicated that the earthquake was a NWW left-lateral strike slip (the local was near the EW strike slip). The displacement was mainly manifested in the east–west direction; we have calculated the displacement was 1.6 m to the east and 2.3 m to the west. Given a certain point as a reference, the maximum displacement was 1.6 m plus 2.3 m, about 4 m, which was consistent with the calculated maximum displacement of 4 m according to the statistical relationship between the maximum slip $D_{\max}$ and the earthquake magnitude $M_{W}$: $M_{W}=6.81+0.781\cdot \log(D_{\max})$ [62]. 

The deformation range in the south of the earthquake fault were significantly larger than that in the north of the earthquake fault, which may indicate that the primary power source causing this earthquake was on the south of the earthquake fault, which was consistent with the knowledge that the Qinghai–Tibet Plateau has been driven northward by the Indian plate. 

Figure 4b showed a clear and intermittent north–south (NS) tensional movement along the main surface rupture, which was consistent with the field investigation [44,53] and the USGS providing the left-lateral strike-slip with a few normal faults. The strike suddenly deflected along the main surface rupture to the west, where the displacement of the north–south direction reached the maximum; we explained that it was likely blocked by some geological body (obstacle body) so that the internal accumulated energy had to be released by the southward change in the rupture strike, and the material was suddenly squeezed southward on the deflecting area. This deflection phenomenon was similar to that of the 2001 Kunlun Mountain Pass West *M*_S_8.1 earthquake [63]. According to the field investigation, the continuity and large surface rupture were found here, including a series of the alternate tensile fissure and squeeze drums, as well as en echelon arrays of shear rupture [48,53]. The above energy release means could also be reasonably explained in the vertical direction. Figure 4c showed that the deformation in the vertical component was alternate subsidence (the maximum reached 0.3 m) and uplift (the maximum reached 0.68 m), which was consistent with the results of the vertical displacement being about 1 m by the unmanned aerial vehicle (UAV) measurement [45]. Where the main surface rupture started from the epicenter to the east, obvious subsidence (Figure 4c) and the small horizontal displacement (Figure 4a) formed some left-stepping tensional cracks; those were consistent with the phenomenon of the small pull-apart basin found in the field [48].

The surface ruptures caused by the Maduo *M*_S_7.4 earthquake were from the epicenter to the east and to the west and had obvious broom-shaped bifurcations at both ends of the rupture. This phenomenon was a typical manifestation of energy release and conformed with the principles of seismology. However, it was a relatively rare deformation feature within the Bayan Har block.

**Figure 5:**
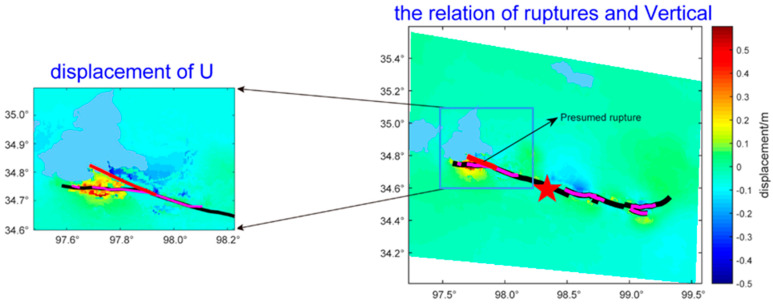
The vertical component displacement of the presumed rupture. (The red line is the presumed rupture that caused the vertical deformation, the purple line is the actual field investigation of the surface rupture, and the black line is the complete rupture according to the actual rupture).

**Figure 6:**
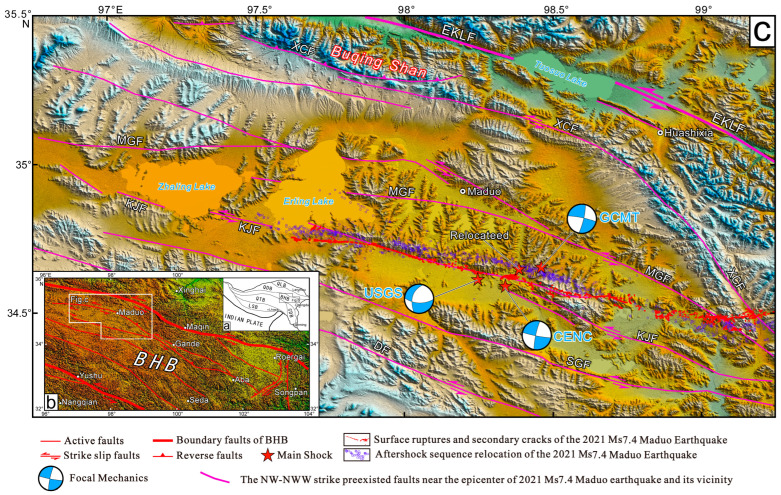
The distribution map of the NW–NWW strike pre-existed faults near the 2021 Maduo *M*_S_7.4 earthquake and its vicinity [55,66,67,68,71,72]. (**a**) is the tectonic unit of the Qinghai-Tibet Plateau. (**b**) is the distribution map of active faults in and around the Bayan Har Block, (**c**) is the specific tectonic location and active faults of the white box in (**b**): BHB—Bayan Har Block, LSB—Lhasa Block, QTB—Qiangtang Block, QDB—Qiadam Block, QLB—Qilian Block. EKLF—East Kunlun Fault, MGF—Maduo-Gande Fault, SGF—South Gande Fault, XCF—Xizangdagou-Changmahe Fault, KJF—Kunlunshankou-Jiangcuo Fault, DF—Dari Fault.

An important finding was that the vertical component had special deformation characteristics at the west end of the main surface rupture, called the near east–west main surface rupture. The boundary between the uplift and subsidence was located on the north side of the near east–west main surface rupture (See Figure 5), while the main surface rupture passed through the uplift areas. This meant that this main surface rupture segment dominated the pure strike-slip movement, while the vertical deformation might have been caused by a pre-existing fault (see the red line in Figure 5). Moreover, the result was consistent with the position of the presumed rupture in the field from Pan et al. [53]. A few deformation traces, but no large-scale surface rupture was obtained from the surface survey [53,64]. Comparing the position with the actual geological map and the geological field survey results from Xiong et al. [65,66,67], a strike-slip fault extending to the east bank of Eling Lake was coincident with the boundary between the subsidence and uplift (See Figure 6). The aftershock sequence relocation also showed that a dense belt of aftershocks occurred along the northwest on the north side of the near east–west main surface rupture [68]. The result provided the evidence for the theoretical hypothesis proposed by Peterson [69] and Livio et al. [70] that the large earthquake could not only cause rupture on the seismogenic fault, but also might trigger pre-existing faults or new faults to produce a surface rupture or weak deformation in areas far away from seismogenic faults.

Combining the deformation result with the field survey, the above analysis results proved the reliability of the calculated three-dimensional deformation, and illustrated the importance of the three-dimensional deformation field for the fine interpretation of the surface rupture and the fault kinematics characteristics.

## 5. Discussion

### 5.1. The Integrating Mutil-Source Data

While the InSAR could not directly provide three-dimensional deformation, the GNSS three-dimensional deformation played an important role in solving the regional three-dimensional deformation field. Compared to the GNSS measurement discreteness, the InSAR measurement was important in spatial scale. The integration of GNSS and InSAR complement each other to maximize their advantages. 

**Figure 7:**
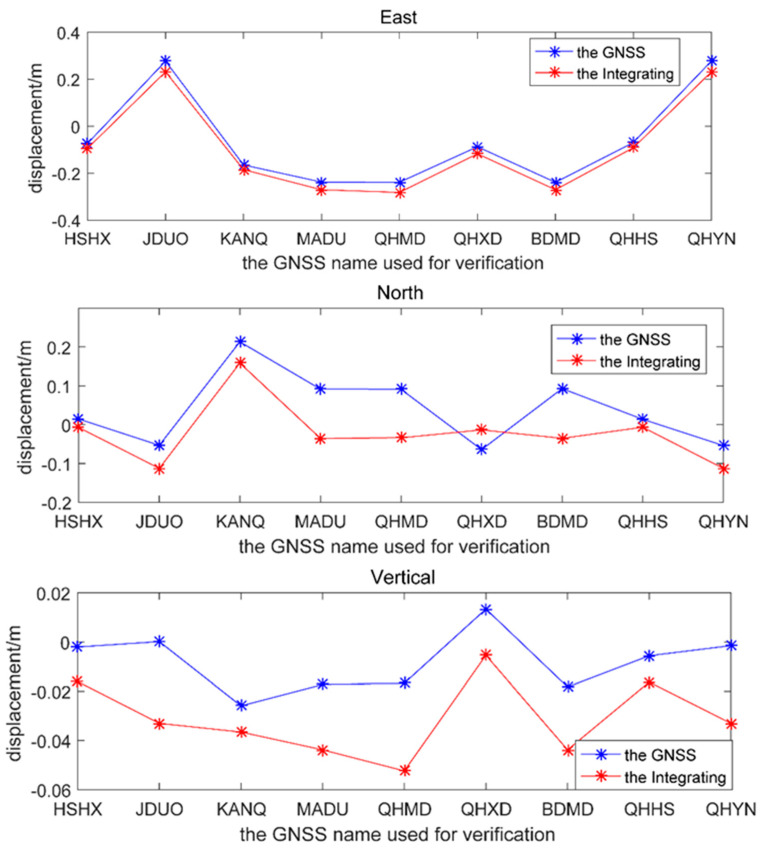
Comparison of GNSS corresponding three-dimensional deformation and the integrated three-dimensional deformation by GNSS and InSAR (the *x*-axis represents the names of the GNSS station for verification, the *y*-axis represents the displacement of the deformation, the red line represents the integrated displacement by the GNSS and InSAR in three directions, and the blue line represents the displacement of the corresponding GNSS stations in three directions).

The differences in root-mean-square errors (RMSE) between calculated three-dimensional deformation and the corresponding GNSS stations were 0.98 cm, 5.64 cm, and 1.37 cm in the east–west, north–south, and vertical directions. The accuracy was better than that of only InSAR measurement, whose RMSEs were 5.2 cm and 12.2 cm in the east–west and north–south directions, but none in the vertical direction [30]. The accuracy of the integrating GNSS and InSAR increased by 81.2% and 53.8% in the east–west direction and north–south direction, respectively, according to the more constrained condition, just as increasing the number of GNSS stations, considering deformation correlation, removing heterogeneous points around the reference points and the variance component estimation weighting. Even if the pixel resolution and the number of SAR satellites in this paper were not as good as those of only InSAR measurement using a multi-satellite and multi-technology approach [30], this result reflected the advantage of integrating the rich high-precision GNSS measurement and the high-spatial resolution InSAR measurement. Since the nadir angle of the SAR satellite was between 20° and 40°, the InSAR LOS direction deformation was more sensitive in the vertical direction than in the horizontal direction, while the InSAR azimuthal direction deformation had no component in the vertical direction and dominated the north–south deformation. Since the GNSS measurement within the correlation distance provided sufficient precision support, the accuracy of the north–south deformation was considerably improved. The accuracy of the north–south direction was still improved, even if the azimuthal direction displacement used only one type of InSAR measurement (MAI_AZI_as) (see Figure 7). The displacement of the GNSS stations was a little larger than that of the integrated result, because GNSS stations used 1~3 days of data before and after the earthquake; however, InSAR observations were from the 21 May, before the earthquake, and the 26 May, after the earthquake in 2021, so the InSAR deformation decreased slightly.

For different types of observations, the calculation precision of the variance component estimation (VCE) weighing was better than the weighted least squares (WLS), which used the observation prior variance as the weight with some uncertainty assumptions, which made it difficult to accurately reflect the observation weight. We took the reciprocal of the observation prior variances within the adaptive window size as the initial weights, then carried out several iterations based on the VCE to obtain the reasonable weights of the observations. The analysis result verified that it was feasible to obtain the high accuracy three-dimensional deformation field by the VCE weighting.

### 5.2. The Geodynamic Characteristic

The Bayan Har block located in the central and eastern regions of the Qinghai–Tibet Plateau is one of the strongest seismically active regions in China nowadays [71]. Its southern and northern boundaries were controlled by two large strike-slip fault zones, the Ganzi–Yushu–Xianshuihe Fault and the East Kunlun Fault, and its eastern boundary consisted of the middle-southern segment of the Longmenshan fault, the Minjiang thrust fault, and the Huya thrust fault. This has been the area of many destructive earthquakes of magnitude 7.0 and above in history [65]. Although a large number of destructive earthquakes had occurred along the boundary of the Bayan Har block, some earthquakes have also occurred within the block, such as the 1947 *M*7¾ earthquake and the 2021 Maduo *M*_S_7.4 earthquake.

The three components interpretation for the co-seismic deformation was helpful to realize the kinematic processing of the seismogenesis structure, and provided the reference for understanding the geological structure and the geodynamics in this region. The three-dimensional deformations showed that the seismogenesis fault had a typical left-lateral strike-slip movement, and the two ends of the main surface rupture had obvious displacement and strike deflections. It was speculated that the fault was blocked by some hard material (obstacle body) during the strike-slip process, which led to the main surface rupture strike southward deflection at the west end and formed two branches at the east end. This was more conducive to energy release in some way. The energy dispersion led to surface distributed deformation [73].

The east–west deformation dominating the NWW strike-slip displacement, had a good indicator of the regional geodynamic environment. The active source of this earthquake might lie in the south of the earthquake fault, which was consistent with the fact that the Qinghai-Tibet Plateau is continuously pushed by the southern Indian plate to the Eurasian plate [74]. The northern boundary and eastern boundary of the Qinghai-Tibet Plateau being blocked by the hard Ordos block and the Sichuan Basin, thus resulted in its southeast escape movement [75]. Several field investigations also indicated that there was a widely distributed deformation range in the south of the earthquake fault [72,76,77,78]. Under the pushing action of the Indian plate, in the south of the Kunlun Maintain Pass-Maduo-Gande fault and in the north of the Qinghai-Tibet Plateau, the material moved faster to SEE than that in the north of the fault, so that the long period of stress accumulation led to this typical strike-slip earthquake.

The vertical deformation in both sides of the main surface rupture (See Figure 4c,f) being in opposite directions, showed that there were some little pivotal movement characteristics in this earthquake.

Intuitively, the southward deflection at the west end and the bifurcation rupture at the east end of the main surface rupture showed that the 2021 Maduo *M*_S_7.4 earthquake caused at least three different strike ruptures. As field investigation from Li et al. [45] and Pan et al. [54] showed, the surface rupture caused by this earthquake had obvious space segmentation. XIAO et al. [79] used the Monte Carlo method and the steepest descent method (SDM) to study the slip distribution of this earthquake and divided the fault into three segments, which also verified the viewpoint of this paper. The result was consistent with the focal mechanism and rupture process research from Deng et al. [80], who believed that the seismogenic fault plane caused by the strong earthquake was not a single plane structure. The above results indicated that the underground structure of the earthquake was complex [81].

**Figure 8:**
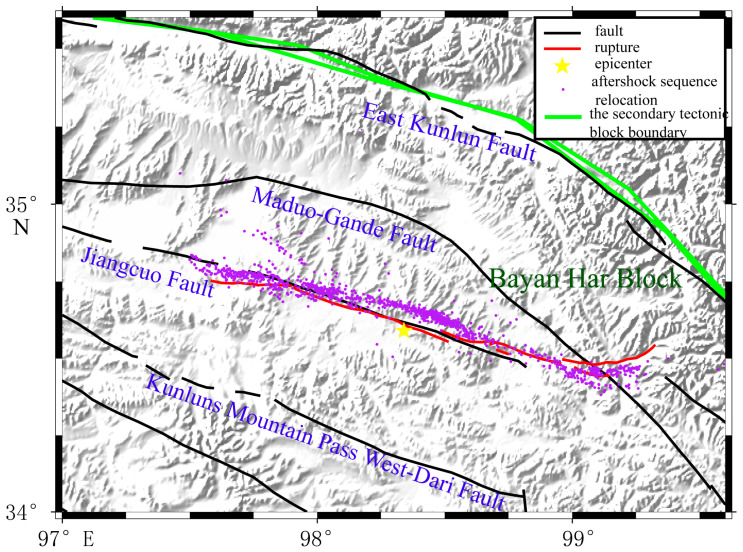
The surrounding faults and the aftershocks relocation of the 2021 Maduo *M*_S_7.4 earthquake: the yellow pentagram represents the location of the epicenter of the 2021 Maduo *M*_S_7.4 earthquake, the purple dot represents the aftershocks sequence relocation of the 2021 Maduo *M*_S_7.4 earthquake, the green line represents the secondary tectonic block boundary, and the black line represents the faults around the epicenter, the red line represents the surface rupture of the 2021 Maduo *M*_S_7.4 earthquake.

The continuous surface rupture was the most obvious in the south of the Eling Lake. The rupture’s geometric distribution was consistent with the bifurcation phenomenon of the aftershock sequence relocation [68] (see Figure 8), which might be blocked by some hard geological body (obstacle body) in the rupture process from the epicenter to both ends. The pre-existing fault could explain the vertical deformation on the north side of the near east–west main surface rupture. In the geological field survey, Pan et al. [54] and Han et al. [82] found that there was no obvious strike-slip dislocation on the north side of the west end of the rupture, but there were several parallel cracks extending to the northwest and disappearing into the Eling Lake. In the research on the late Quaternary activity of the related faults in the north of the Bayan Har block, we also found that there were several NW–NWW strike-slip active faults near main surface rupture of this earthquake [67] (see Figure 6).

The surface rupture and deformation characteristics of the Maduo earthquake were significantly different from those of several strike-slip earthquakes that had occurred in the Qinghai–Tibet Plateau in the past, and this indicates the complexity of the earthquake. For example, the main surface rupture of the 2001 Kunlun Mountain Pass West *M*_S_8.1 earthquake [63], located on the northern boundary of the Bayan Har block, was somewhat similar to that of the Maduo earthquake; it also showed strike deflection at the west end of the rupture. However, the rupture process of the 2010 Yushu *M*_S_7.1 earthquake [83], which occurred on the southern boundary of the Bayan Har block, was a typical unilateral rupture, and the deformation characteristic was significantly different from that of the Maduo earthquake in the vertical direction, which was uplift and subsidence, but with no obvious alternation on both sides along the surface rupture of the Yushu earthquake.

**Figure 9:**
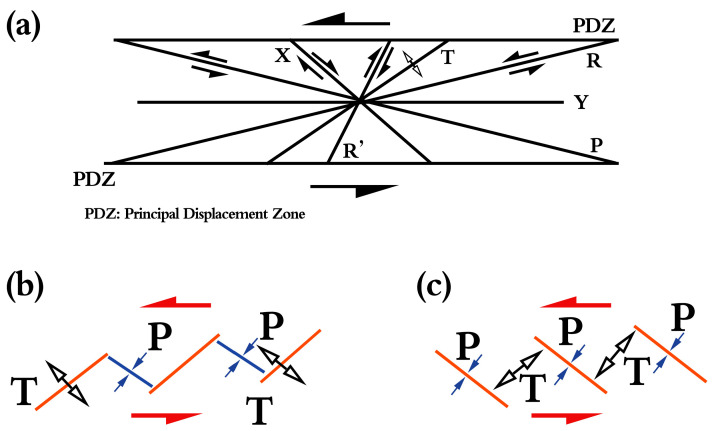
The Riedel shear in strike-slip fault zone. (**a**) An idealized Riedel shear zone is composed of six principal elements, which are X shear and Y shear faults, Riedel (R) and R′ conjugate shears, T tensional fractures, and P shears, which are all oriented at specific angles with respect to the general trend of the shear zone. (**b**) Mechanism of the right-stepping in strike-slip fault zone. (**c**) Mechanism of the right-stepping in strike-slip fault zone (modified from Li et al. [85]): The blue arrows represent the compressional tectonics, and the white arrows represent the extensional tectonics. The red lines represent the cracks, the bule lines represent squeeze effect on the plane.

In the surface rupture, we could see some left-stepping tensional cracks and pull-apart basins; the mechanism of these phenomena was caused by the Riedel shear. The major strike-slip faults, which may be thousands of kilometers long and tens of kilometers wide, are in domains of simple shear, and the displacement can be monitored in hundreds of kilometers along a fault. Within the domain of simple shear, the most recently active strand might be a zone of active faulting that was only a few meters wide. The simple shear had a donoclinic symmetry of strain because it was rotational, and a greater variety of structure forms in simple shear than in pure shear [84]. The en echelon arrays composed of earthquake faults, extension fractures, and pressure ridges are very common in a strike-slip fault zone. Left-stepping and right-stepping in a left-lateral strike-slip fault zone is generally derived from extensional tectonics and compressional tectonics, respectively (see Figure 9a,b). Geometrically, an idealized Riedel shear zone was composed of six principal elements (Figure 9c), which were X shear and Y shear faults, Riedel (R) and R′ conjugate shears, T tensional fractures, and P shears, which were all oriented at specific angles with respect to the general trend of the shear zone [85]. The co-seismic Riedel shear structures revealed a left-lateral strike-slip sense for the seismogenic fault of the 2021 Maduo *M*_S_7.4 earthquake [86]. The focal mechanism data for the Maduo earthquake showed two nodal faults trending nearly EW and NS, which was in accordance with the field investigations [75,76,77,78,79,80,81,82,83,84,85,86,87]. The Riedel shear model primarily controlled the earthquake surface ruptures of a strike-slip earthquake and dominated the formation and evolution of strike-slip faults from a wider perspective.

The calculated three-dimensional deformation field was in agreement with several geological field surveys [44,45,53,66,67,68,72,75,76,77,80,81,82,88,89,90,91,92,93,94,95,96,97]. There was no obvious surface rupture near the epicenter, while there were some large-scale forked-broom-shaped co-seismic surface ruptures on both ends of the rupture. This verified the research result [84] that the surface deformation and the rupture characteristics with the multiple bifurcation, tail folding, and spalling at the ends of the rupture, were very different from those of the main strike-slip section of the strike-slip fault.

Combining the surface rupture field investigation with the regional three-dimensional deformation field by integrating the GNSS and InSAR, we considered that the 2021 Maduo *M*_S_7.4 earthquake was a typical left-lateral strike-slip earthquake; the revealed kinematic characteristics of the seismogenic fault showed that there was a relatively obvious dip-slip component at the ends of the rupture.

In the inversion of the three-dimensional co-seismic deformation field, we also made the important discovery that there was the obvious left-lateral strike-slip deformation but no vertical deformation at the west end of the main surface rupture, while the pre-existing fault dominated the vertical deformation. This discovery was an important supplement to the co-seismic deformation characteristics obtained from the field investigation. It provided an important reference for further exploring this earthquake’s deformation mechanism and regional geodynamics.

## 6. Conclusions

The deformation correlation between the reference point and the points involved in the solution was fully considered, and it was taken as the decorrelation distance range for selecting the points used. An adaptive method was proposed to obtain a high accuracy regional three-dimensional deformation field. Taking the 2021 Maduo MS7.4 earthquake as an example, based on the elasticity theory, we established the accurate regional co-seismic three-dimensional deformation field by effectively and rationally integrating GNSS and InSAR, and gave the crustal dynamics and geological explanation of the three-dimensional co-seismic deformation field. The conclusions could be drawn as follows.

(1) The accuracy obtained by integrating GNSS and InSAR increased by 81.2% and 53.8% in the east–west direction and north–south direction, respectively. Additional constraints were helpful to obtain a high accuracy regional three-dimensional deformation field, such as increasing the GNSS stations, considering deformation correlation, removing heterogeneous points around the reference points, and the variance component estimation weighting.
(2) The spatial position and the left-lateral strike-slip motion of the earthquake were consistent with those of the Jiangcuo section of the NWW strike of the Kunlun Mountain Pass–Jiangcuo fault in the south of the East Kunlun Fault. The mechanism of the left-lateral left-stepping was caused by the Riedel shear, which dominated the formation and evolution of strike-slip faults.
(3) The maximum slip of the earthquake was about 4 m, which was difficult to investigate in the geological field survey due to the lack of reliable markers, and the slip was consistent with the empirical statistical results.
(4) The ruptures started from the epicenter to both ends and formed bifurcations, which were rarely found in the same type of previous earthquakes that occurred in the Qinghai–Tibet Plateau.
(5) The pre-existing fault controlled the vertical deformation at the north side of the near east–west surface rupture, which only dominated the east–west strike-slip. The three-dimensional deformation was consistent with the surface rupture trace. This provided direct evidence for the theoretical hypothesis that large earthquakes can not only produce a surface rupture on seismogenic faults, but also trigger pre-existing faults or new faults to produce surface ruptures or weak deformation in areas far from seismogenic faults.

We recognized that the new seismic surface rupture did not necessarily completely control the distribution of seismic deformation. For example, in the western end of the surface rupture of the Maduo earthquake, the strike-slip deformation was distributed along the new near east–west surface rupture, while the vertical deformation was controlled by the pre-existing structure in the northwest direction.

The deformation distribution range in the south of the fault was wider than that in the north, which might indicate that the physical properties of the geological bodies were different on both sides of the fault. The northward extrusion of the Indian plate led to the formation of large strike-slip faults in the Qinghai–Tibet Plateau, and these faults constituted the boundary of the active block. The epicenter was located on the south of the East Kunlun fault, which was the northern boundary of the Bayan Har block. The co-seismic deformation field indicated that the thrusting effect from the southern Indian plate was transmitted to the epicenter. The block in the south of the earthquake fault moved faster in the SEE direction than the block in the north. In future, we would obtain the displacement distribution on both sides of the surface rupture through a series of deformation profiles, and compare it with the field investigation to further discuss the changing trend of deformation and its mechanism. In addition, the relationship between the earthquake fault and the East Kunlun fault, and the relationship between the new rupture and the pre-existing fault are topics to be further studied.

This series of results formed an important supplement to the field survey and provided a reference for the risk assessment of geological hazards [98] in the area of interest. It provided a novel idea for the integration of multi-source measurement technologies to improve seismic deformation monitoring. However, it should be noted that it will be a modeling challenge for the real earth.

## Figures and Tables

**Table 1:** The information of the used SAR satellite data.

Orbit	Acquisition Date of the Master Image	Acquisition Date of the Slave Image	Imaging Mode	Wavelength /cm	Revisit Period /d
Ascending (T99)	20 May 2021 (Sentinel-1A)	26 May 2021 (Sentinel-1B)	IW	5.6	6
Descending (T106)	20 May 2021 (Sentinel-1A)	26 May 2021 (Sentinel-1B)	IW	5.6	6

## Data Availability

The other data used to support the findings of this study are included within the article.

## References

[1] Jiang Z.S., Ma Z.J., Niu A.F., Zhang X.L., Wang S.X., Chen B. (2003). Approaches and Preliminary results of Crust movement researches based on the GPS in China. Earth Sci. Front..

[2] Guemundsson S. (2000). Crustal Deformation Mapped by Combined GPS and InSAR.

[3] Gudmundsson S., Sigmundsson F. (2002). Three-Dimensional Surface Motion Maps Estimated from Combined Interferometric Synthetic Aperture Radar and GPS Data. J. Geophys. Res..

[4] Samsonov S.V., Tiampo K.F., Rundle J., Li Z.H. (2007). Application of InSAR-GPS Optimization for Derivation of Fine Scale Surface Motion Maps of Southern Califotnia. IEEE Trans. Geosci. Remote Sens..

[5] Samsonov S.V., Tiampo K.F. (2006). Analytical optimization of DInSAR and GPS dataset for derivation of three-dimensional surface motion. IEEE Geosci. Remote Sens. Lett..

[6] Hu J., Li Z.W., Sun Q., Zhu J.J., Ding X.L. (2012). Three-dimensional surface displacements from InSAR and GPS measurements with variance component estimation. IEEE Geosci. Remote Sens. Lett..

[7] Hu J., Li Z.W., Zhu J.J., Ding X.L. (2013). Measuring three-dimensional surface displacements from combined InSAR and GPS data based on BFGS method. Chin. J. Geophys..

[8] Hu J., Li Z.W., Ding X.L., Zhu J.J., Sun Q. (2013). Derivation of 3-D coseismic surface displacement field for the 2011 Mw9.0 Tohoku-Oki earthquake from InSAR and GPS measurements. Geophys. J. Int..

[9] Elisabeth S., Stephane D., Jordan B., Laurent P., Laurent M., Joelle N.D. (2014). Combination of INSAR and GNSS measurements for ground displacement monitoring. Procedia Technol..

[10] Leijen F.J., Samiei-Esfahany S., Marel H.V., Hanssen R.F. A standardized approach for the integration of geodetic data for deformation analysis. Proceedings of the 2017 IEEE International Geoscience and Remote Sensing Symposium (IGARSS).

[11] He X.F., Shi G.Q., Xiao R.Y. (2015). Integration of GPS and InSAR for 3D Deformation Monitoring Based on Ant Colony Optimization. J. Tonfji Univ. (Nat. Sci.).

[12] Cao H.K., Zhao L.H., Zhang Q., Qu W., Nie J.L. (2018). Ascending and Desending Orbits InSAR-GPS Data Fusion Method with Additional Systematic Parameters for Three-Dimensional Deformation Field. Ceomatics Inf. Sci. Wuhan Univ..

[13] Zhu J.J., Yang Z.F., Li Z.W. (2019). Recent progress in retrieving and predicting mining-induced 3D displace-ments using InSAR. Acta Geod. Et Cartogr. Sin..

[14] Béjar-Pizarro M., Guardiola-Albert C., García-Cárdenas R.P., Herrera G., Barra A., López Molina A., Tessitore S., Staller A., Ortega-Becerril J.A., García-García R.P. (2016). Interpolation of GPS and Geological Data Using InSAR Deformation Maps: Method and Application to Land Subsidence in the Alto Guadalentín Aquifer (SE Spain). Remote Sens..

[15] Hanssen R.F. (2001). Radar Interferometry: Data Interpretation and Error Analysis.

[16] Guglielmino F., Nunnari G., Puglisi G., Spata A. (2011). Simultaneous and Integrated Strain Tensor Estimation from Geodetic and Satellite Deformation Measurements to Obtain Three-Dimensional Displacement Maps. IEEE Trans. Geosci. Remote Sens..

[17] Guglielmino F., Bignami C., Bonforte A., Briole P., Obrizzo F., Puglisi G., Stramondo S., Wegmuller U. (2011). Analysis of satellite and in situ ground deformation data integrated by the SISTEM approach: The 3 April 2010 earthquake along the Pernicana fault(Mt.Etna-Italy) case study. Earth Planet Sci. Lett..

[18] Gugliemino F., Anzidei M., Briole P., Elias P., Puglisi G. (2013). 3D Displacement maps of the 2009 L’Aquila earthquake (Italy) by applying the SISTEM method to GPS and DInSAR data. Terra Nova.

[19] Luo H.P., Chen T. (2016). Three-Dimensional Surface Displacement Field Associated with the 25 April 2015 Gorkha, Nepal, Earthquake: Solution from Integrated InSAR and GPS Measurements with an Extended SISTEM Approach. Remote Sens..

[20] Xiong L.Y., Xu C.J., Liu Y., Zhao Y.W. (2022). Three-dimensional displacement field of the 2021 Mw8.8 Maule earthquake from GPS and InSAR data with the improved ESISTEM-VCE method. Front. Earth Sci..

[21] Wang X., Liu G., Yu B., Dai K., Zhang R., Ma D., Li Z. (2015). An integrated method based on DInSAR, MAI and displacement gradient tensor for mapping the 3D coseismic deformation field related to the 2011 Tarlay earthquake (Myanmar). Remote Sens. Environ..

[22] Wang Z.W. (2019). Research on resolving of three-dimensional displacement from multi-source InSAR data. Acta Geod. Cartogr. Sin..

[23] Zhu J.J., Li Z.W., Hu J. (2017). Research progress and methods of InSAR for deformation monitoring. Acta Geod. Cartogr. Sin..

[24] Gao M.L., Gong H.L., Chen B.B., Zhou C.F., Si Y. (2017). Review of Three-dimensional Surface Deformation Acquisition from InSAR Measurements and Its Application. Bull. Surv. Mapp..

[25] Liu J.H., Hu J., Li Z.W., Zhu J.J., Sun Q., Gan J. (2018). A method for measuring 3-D surface deformations with InSAR based on strain model and variance component estimation. IEEE Trans. Geosci. Remote Sens..

[26] Gan J., Hu J., Li Z.W., Yang C.J., Liu J.H., Sun Q., Zheng W.J. (2018). Mapping three-dimensional co-seismic surface deformations associated with the2015 MW7.2 Murghab earthquake based on InSAR and characteristics of crustal strain. Sci. China Earth Sci..

[27] Yuan S., He P., Wen Y.M., Xu C.J. (2020). Integrated InSAR and strain tensor to estimate three-dimensional coseismic displacements associated with the 2016 Mw7.0 Kumamoto earthquake. Chin. J. Geophys..

[28] Hu J., Liu J., Li Z., Zhu J., Wu L., Sun Q., Wu W. (2021). Estimating three-dimensional co-seismic deformations with the SM-VCE method based on heterogeneous SAR observations: Selection of homogeneous points and analysis of observation combinations. Remote Sens. Environ..

[29] Liu J.H., Hu J., Li Z.W., Zhu J.J. (2021). Estimation of 3D coseismic deformation with InSAR: An improved SM-VCE method by window optimization. Acta Geod. Et Cartogr. Sin..

[30] Liu J.H., Hu J., Li Z.W., Ma Z., Wu L., Jiang W., Feng G., Zhu J.J. (2022). Complete three-dimensional coseismic displacements due to the 2021 Maduo earthquake in Qinghai Province, China from Sentinel-1 and ALOS-2 SAR images. Sci. China Earth Sci..

[31] Gabriel A.K., Goldstein R.M., Zebker H.A. (1989). Mapping small elevation changes over large areas: Differential radar interferometry. J. Geophys. Res. Solid Earth.

[32] Bechor N.B.D., Zebker H.A. (2006). Measuring two-dimensional movements using a single InSAR pair. Geophys. Res. Lett..

[33] Michel R., Avouac J.P., Taboury J. (1999). Measuring ground displacements from SAR amplitude images: Application to the landers earthquake. Geophys. Res. Lett..

[34] Grandin R., Klein E., Métois M., Vigny C. (2016). 3D displacement field of the 2015 Mw8. 3 Illapel earthquake (Chile) from across- and along-track Sentinel-1 TOPS interferometry. Geophys. Res. Lett..

[35] Okada Y. (1985). Surface deformation due to shear and tensile faults in a half-space. Seismol. Soc. Am..

[36] Hu J. (2012). Theory and Method of Estimating Three-Dimensional Displacement with InSAR Based on the Modern Surveying Adjustment.

[37] Xu P., Liu Y., Shen Y., Fukuda Y. (2007). Estimability analysis of variance and covariance components. J. Geod..

[38] Xu P., Shen Y., Fukuda Y., Liu Y. (2006). Variance component estimation in linear inverse ill-posed models. J. Geod..

[39] Cui X.Z., Yu Z.C., Tao B.Z. (2009). Generalized Surveying Adjustment (New ER).

[40] Nie Y., Shen Y., Pail R., Chen Q. (2022). Efficient variance component estimation for large-scale least-squares problems in satellite geodesy. J. Geod..

[41] Li X.B., Wang X.Y., Chen Y.L. (2022). InSAR Atmospheric Delay Correction Model Integrated from Multi-Source Data Based on VCE. Remote Sens..

[42] Xu C.J., Deng C.Y., Zhou L.X. (2016). Coseismic slip distribution inversion method based on the variance component estimation. Geomat. Inf. Sci. Wuhan Univ..

[43] Hua J., Zhao D.Z., Shan X.J., Qu C.Y., Zhang Y.F., Gong W.Y., Wang Z.J., Li C.L., Li Y.C., Zhao L. (2021). Coseismic deformation field, slip distribution and coulomb stress disturbance of the 2021 Mw7.3 Maduo Earthquake using sentinel-1 InSAR observation. Seismol. Geol..

[44] Deng Q.D., Zhang P.Z., Ran Y.K., Yang X.P., Min W., Chen L.C. (2003). Active tectonics and earthquake activities in China. Earth Sci. Front..

[45] Li Z.M., Li W.Q., Li T., Xu Y.R., Su P., Guo P., Sun H.Y., Ha G.H., Chen G.H., Yuan Z.D. (2021). Seismogenic fault and coseismic surface deformation of the Maduo Ms7.4 earthquake in Qinghai, China: A quick report. Seismol. Geol..

[46] Wang W.X., Shao Y.X., Yao W.Q. (2022). Rapid extraction of features and indoor reconstruction of 3D structures of Madoi Mw7.4 earthquake surface rupture based on photogrammetry method. Seismol. Geol..

[47] Ren J.J., Xu X.W., Zhang G.W., Wang Q.X., Zhang Z.W., Gai H.L., Kang W.J. (2022). Co-seismic surface ruptures, slip distribution, and 3D seismogenic fault for the 2021 Mw 7.3 Maduo earthquake, central Tibetan Plateau, and its tectonic implications. Tectonophysics.

[48] Li Z.C., Ding K.H., Zhang P., Wen Y.M., Zhao L.J., Chen J.F. (2021). Co-seismic Deformation and Slip Distribution of 2021 Mw 7.4 Madoi Earthquake from GNSS Observation. Geomat. Inf. Sci. Wuhan Univ..

[49] Wang D.J., Wang D.Z., Zhao B., Li Y., Zhao L.J., Wang Y.B., Nie Z.S., Qiao X.J., Wang Q. (2022). 2021 Qinghai Madoi MW7.4 earthquake coseismic deformation field and fault-slip distribution using GNSS observations. Chin. J. Geophys..

[50] Shu C.Z. (2022). InSAR 3D Coseismic and Postseismic Deformation of Maduo Mw7.4 and Inversion of Fault Slip Distrobution.

[51] Yu P.F., Xiong W., Chen W., Qiao X.J., Wang D.J., Liu G., Zhao B., Nie Z.S., Li Y., Zhao L.J. (2022). Slip model of the 2021 M_S_7.4 Madoi earthquake constrained by GNSS and InSAR coseismic deformation. Chin. J. Geophys..

[52] Gao Z.Y., Li Y.C., Shan X.J., Zhu C.H. (2021). Earthquake Magnitude Estimation from High-Rate GNSS Data: A Case Study of the 2021 Mw 7.3 Maduo Earthquake. Remote Sens..

[53] Yang J.Y., Sun W.K., Hong S.Y., Yuan Z.Y., Li Y., Chen W., Meng G.j. (2021). Coseismic deformation analysis of the 2021 Qinghai Madoi M7.4 earthquake. Chin. J. Geophys..

[54] Pan J.W., Bai M.K., Li C., Liu F.C., Li H.B., Liu D.L., Chevalier M.L., Wu K.G., Wang P., Lu H.J. (2021). Cosesimic surface rupture and Seismogenic structure of the 2021-05-22 Maduo (Qinghai) Ms7.4 earthquake. Acta Geol. Sin..

[55] Wang Y.B., Li Y., Cai Y., Jiang L.J., Shi H.B., Jiang Z.S., Gan W.J. (2022). Coseismic displacement and slip distribution of the 2021 May 22, Ms7.4 Madoi earthquake derived from GNSS observations. Chin. J. Geophys..

[56] Hu J., Li Z.W., Zhu J.J., Ren X.C., Ding X.L. (2010). Inferring three-dimensional surface displacement field by combining SAR interferometric phase and amplitude information of scending and descending orbits. Sci. China Earth Sci..

[57] Zhang Q., Zhao C.Y., Ding X.L., Chen Y.Q., Wang L., Huang G.W., Yang C.S., Ding X.G., Ma J. (2009). Research on recent characteristics of spatio temporal evolution and mechanism of Xi’an land subsidence and ground fissure by using GPS and InSAR techniques. Chin. J. Geophys..

[58] Farr T.G., Rosen P.A., Caro E., Crippen R., Duren R., Hensley S., Alsdorf D. (2007). The Shuttle Radar Topography Mission. Rev. Geophys..

[59] Parsons B., Wright T., Rowe P., Andrews J., Jackson J., Walker R., Khatib M., Talebian M., Bergman E., Engdahl E.R. (2006). The 1994 Sefidabeh (eastern Iran) earthquakes revisited: New evidence from satellite radar interferometry and carbonate dating about the growth of an active fold above a blind thrust fault. Geophys. J. Int..

[60] Sudhaus H., Jónsson S. (2011). Source model for the 1997 Zirkuh earthquake (MW = 7.2) in Iran derived from JERS and ERS InSAR observations. Geophys. J. Int..

[61] Zhao T., Wang Y., Ma J., Shao R.T., Xu Y.F., Hu J. (2021). Relocation and focal mechanism solution of the 2021 Maduo, Qinghai Ms7.4 earthquake sequence. Seismol. Geol..

[62] Wells D.L., Coppersmith K.J. (1994). New empirical relationships among magnitude, rupture length, rupture width, rupture area, and surface displacement. Bull. Seismol. Soc. Am..

[63] Dong R.D., Yao Q., Shi H.Q., Wang Q. (2022). A Unified Model of Spatiotemporal Rupture Process for the 2001 Weast of Kunlun Mountain Pass Earthquake. Earthq. Res. China.

[64] Wang W.M., He J.K., Wang X., Zhou Y., Hao J.L., Zhao L.F., Yao Z.X. (2022). Rupture process models of the Yangbi and Maduo earthquakes that struck the eastern Tibetan Plateau in May 2021. Sci. Bull..

[65] Xiong R.W. (2010). Study on the Activity of Maduo-Gande Fault.

[66] Xiong R.W., Ren J.W., Zhang J.L., Yang P.X., Li Z.M., Hu C.Z., Chen C.Y. (2010). Late quaternary active characteristics of the Gande segment in the Maduo-Gande fault zone. Earthquake.

[67] Xiong R.W. (2013). Late Quaternary Active Characteristics of the Maduo-Gande Fault Zone in Bayankala Block.

[68] Wang W., Fang L., Wu J., Tu H., Chen L., Lai G., Zhang L. (2021). Aftershock sequence relocation of the 2021 Ms7.4 Maduo Earthquake, Qinghai, China. Sci. China Earth Sci..

[69] Pertersen M.D., Dawson T.E., Chen R., Cao T.Q., Wills C.J., Schwartz D.P., Frankel A.D. (2011). Fault displacement hazard for strike-slip faults. Bull. Seismol. Soc. Am..

[70] Livio F., Serval L., Gurpinar A. (2016). Locating distirbuted faulting: Contributions from InSAR imaging to probabilistic fault displacement hazard analysis (PFDHA). Quat. Int..

[71] Zhang Y.M., Li M.F., Meng Y.Q., Wu S.X., Cai C.X. (1996). Research on fault activities and their seismogeological implication in Bayankala Mountain area. Res. Act. Fault..

[72] Yuan Z., Li T., Su P., Sun H., Ha G., Guo P., Chen G., Jobe J.T. (2022). Large surface rupture gaps and low surface fault slip of the 2021 Mw 7.4 Maduo earthquake along a low-activity strike-slip fault, Tibetan Plateau. Geophys. Res. Lett..

[73] Song X.H., Wang S.J., Pan S.Z., Song J.J. (2021). Deep seismotectonic environment of the 2021 Madoi Ms7.4 earthquake. Seismol. Geol..

[74] Deng Q.D., Cheng S.P., Ma J., Du P. (2014). Seismic activities and earthquake potential in the Tibetan plateau. Chin. J. Geophys..

[75] Li C.J. (2021). Lithospheric Deformation Characteristics and Related Mechanisms in SE Tibetan Plateau.

[76] Yuan J.Y., Wang Y.L., Zhan B.L., Yuan X.M., Wu X.Y., Ma J.J. (2022). Comprehensive investigation and analysis of liquefaction damage caused by the Ms7.4 Maduo earthquake in 2021 on the Tibetan Plateau, China. Soil Dyn. Earthq. Eng..

[77] Yao W.Q., Wang Z.J., Liu J., Liu X.L., Han F.L., Shao Y.X., Wang W.X., Xu J., Qin K.X., Gao Y.P. (2022). Discussion on coseismic surface rupture length of the 2021 Mw7.4 Madoi earthquake, Qinghai, China. Seismol. Geol..

[78] Lv M.M., Chang L.J., Lu L.Y., Liu J.D., Wu P.P., Guo H.L., Cao X.L., Ding Z.F. (2022). Focal mechanism solutions of the aftershocks of the 2021 Qinghai Madoi MS7.4 earthquake and its seismogenic structure characteristics. Chin. J. Geophys..

[79] Xiao L.H., Zheng R., Zou R. (2022). Co-seismic Slip Distribution of THE 2021 Mw7.4 Maduo, Qinghai Earthquake Estimated from InSAR and GPS Measurements. J. Earth Sci..

[80] Deng W.Z., Liu J., Yang Z.G., Sun L., Zhang X.M. (2022). Preliminary analysis for rupture process of the May 22th, 2021, Madoi (Qinghai) Ms7.4 earthquake. Seismol. Geol..

[81] Zhan Y., Liang M.J., Sun X.Y., Huang F.P., Zhao L.Q., Gong Y., Han J., Li C.X., Zhang P.Z., Zhang H.P. (2021). Deep structure and seismogenic pattern of the 2021.5.22 Madoi (Qing hai) Ms7.4 earthquake. Chin. J. Geophys..

[82] Han L.F., Liu J., Yao W.Q. (2022). Detailed mapping of the surface rupture near the epicenter segment of the 2021 Madoi M_W_7.4 earthquake and discussion on distributed rupture in the step-over. Seismol. Geol..

[83] Xie W.B., Hu J.W., Zuo X.Q., Zhang Y.M., Liu Y.Z. (2019). Analysis of 3D deformation field caused by Yushu Mw7. 1 earthquake based on InSAR technology. Geotech. Investig. Surv..

[84] Sylvester A.G. (1988). Strike-slip faults. Geol. Soc. Am. Bull..

[85] Li H.B., Pan J.W., Lin A., Sun Z.M., Liu D.L., Zhang J.J., Li C.L., Liu K., Chevalier M.L., Yun K. (2016). Coseismic surface ruptures associated with the 2014 *M*_W_ 6.9 yutian earthquake on the Altyn Tagh fault, Tibetan Plateau. Bull. Seismol. Soc. Am..

[86] Ren J.J., Zhang Z.W., Gai H.L., Kang W.J. (2021). Typical Riedel shear structures of the coseismic surface rupture zone produced by the 2021 *M*_W_ 7.3 Maduo earthquake, Qinghai, China, and the implications for seismic hazards in the block interior. Nat. Hazards Res..

[87] Earthquake Agency of Xinjiang Uygur Autonomous Region (1985). The Fuyun Earthquake Fault Zone.

[88] Xu Z.G., Liang S.S., Zhang G.W., Liang J.H., Zou L.Y., Li X.M., Chen Y.H. (2021). Analysis of seismogenic structure of Madoi, Qinghai MS7.4 earthquake on May 22, 2021. Chin. J. Geophys..

[89] Jin Z., Fialko Y. (2021). Coseismic and early postseismic deformation due to the 2021 M7.4 Maduo (China) earthquake. Geophys. Res. Lett..

[90] He L.J., Feng G.C., Wu X.X., Lu H., Xu W.B., Wang Y.D., Liu J.H., Hu J., Li Z.W. (2021). Coseismic and early postseismic slip models of the 2021 Mw 7.4 Maduo earthquake (Western China) estimated by space-based geodetic data. Geophys. Res. Lett..

[91] Chen K.J., Avouac J.P., Geng J.H., Liang C.R., Zhang Z.G., Li Z.C., Zhang S.P. (2022). The 2021 Mw7.4 Madoi earthquake: An archetype bilateral slip-pulse rupture arrested at a splay fault. Geophys. Res. Lett..

[92] Zhao D., Qu C., Chen H., Shan X., Song X., Gong W. (2021). Tectonic and geometric control on fault kinematics of the 2021 Mw7.3 Maduo (China) earthquake inferred from interseismic, coseismic, and postseismic InSAR observations. Geophys. Res. Lett..

[93] Pan J.W., Li H.B., Chevalier M.L., Tapponnier P., Bai M.K., Li C., Liu F.C., Liu D.L., Wu K.G., Wang P. (2022). Co-seismic rupture of the 2021, Mw7.4 Maduo earthquake (northern Tibet): Short-cutting of the kunlun fault big bend. Earth Planet. Sci. Lett..

[94] Shao Y.X., Liu Z.J., Gao Y.P., Wang W.X., Yao W.Q., Han F.L., Liu Z.J., Zou X.B., Wang Y., Li Y.S. (2022). Co-seismic displacement measurement and distributed deformation characterization: A case of 2021 Mw7.4 Madoi earthquake. Seismol. Geol..

[95] Liu X.L., Xia T., Liu J. (2022). Distributed characteristics of the surface deformation associated with the 2021 MW7.4 Madoi earthquake, Qinghai, China. Seismol. Geol..

[96] Chen H., Qu C.Y., Zhao D.Z., Ma C., Shan X.J. (2021). Rupture kinematics and coseismic slip model of the 2021 Mw7.3 Maduo (China) earthquake: Implications for the seismic hazard of the kunlun fault. Remote Sens..

[97] Zheng A., Yu X.W., Qian J.Q., Liu X.G., Zhang W.B., Chen X.F., Xu W.B. (2023). Cascading rupture process of the 2021 Maduo, China earthquake revealed by the joint inversion of seismic and geodetic data. Tectonophysics.

[98] Zhu M., Chen F.L., Fu B.H., Chen W.K., Qiao Y.F., Shi P.L., Zhou W., Lin H., Liao Y.A., Gao S. (2023). Earthquake-induced risk assessment of cultural heritage based on InSAR and seismic intensity: A case study of Zhalang temple affected by the 2021 Mw 7.4 Maduo (China) earthquake. Int. J. Disaster Risk Reduct..

